# Evaluation in the Real World: Decision Points and Rationales in Creating A Rigorous Study Designed to Convey Ecologically Valid Findings

**DOI:** 10.1002/ajcp.12485

**Published:** 2020-12-16

**Authors:** Cris M. Sullivan, Danielle Chiaramonte, Gabriela López‐Zerón, Katie Gregory, Linda Olsen

**Affiliations:** ^1^ Michigan State University East Lansing MI USA; ^2^ Yale University New Haven CT USA; ^3^ Washington State Coaliltion Against Domestic Violence Seattle WA USA

**Keywords:** Program evaluation, Domestic violence, Intimate partner violence, Randomized, Ecological validity

## Abstract

Rigorously evaluating community‐based interventions for multiply marginalized populations is fraught with challenges under the best of circumstances. This manuscript describes the methodology chosen to evaluate an innovative model designed to help survivors of intimate partner violence obtain safe and stable housing. We justify the choice of evaluation design from a community psychology perspective and detail why we believe the multi‐method, multi‐source design, that also focuses on social context, will maximize ecological validity and, therefore, propel the scale‐up of the intervention if it is found to be effective. Longitudinal data are being collected from program recipients over time, the advocates who worked with them, agency service records, and monthly documentation of agency resources on hand that can impact services provided. Special attention is focused on capturing contextual information that can impact program success. While randomized control trials are still too often heralded as “the gold standard” for measuring intervention effectiveness, we maintain that the current design, which was developed in partnership with key community stakeholders, holds more promise when evaluating many community‐based programs.

## Highlights

- Pragmatic evaluations, designed collaboratively with community partners, can increase ecological validity.
- Community psychologists can learn from concrete examples of pragmatic, real‐world evaluations.
- This manuscript describes the development of a multi‐method, longitudinal quasi‐experimental evaluation.
- The evaluation design includes a focus on how social context can impact program outcomes.

## Introduction

Evaluating community‐based interventions is complicated under the best of circumstances and is especially challenging when the programs serve extremely vulnerable populations. Evaluators have to weigh design trade‐offs between internal validity and external validity, and community psychologists are especially interested in maximizing ecological validity—the extent to which findings are drawn from and applicable to real‐world settings. This manuscript describes a longitudinal, multi‐method, multi‐source design, developed in partnership with key community stakeholders, as one example of community‐based research intended to maximize ecological validity and, therefore, propel the scale‐up of an intervention if it is found to be effective.

Community‐based programs working with multiply marginalized populations tend to be dynamic and complex, and do not lend themselves to “textbook” evaluation methods that fail to consider context and real‐world factors (Campbell, Patton, & Patrizi, 2003; Goodman, Epstein, & Sullivan, 2018; Schwandt, 2015). While randomized control trials (RCTs) are excellent designs to determine intervention outcomes examined under highly controlled conditions, they do not capture the complexities inherent within community services offered to vulnerable populations (Crane et al., 2019; Goodman et al., 2018; Sanson‐Fisher, Bonevski, Green, & D'Este, 2007). In order to evaluate these programs in a manner that will produce ecologically valid findings that are feasible, actionable, and relevant to community stakeholders, researchers need to employ pragmatic evaluation approaches that include a focus on community context (Glasgow, 2013; Glasgow & Chambers, 2012). Glasgow and colleagues (Glasgow, 2013; Glasgow, Magid, Beck, Ritzwoller, & Estabrooks, 2005) recommend that pragmatic evaluations (a) prioritize outcomes that are important to community stakeholders, (b) include multiple, diverse settings to enhance generalizability, (c) minimize exclusion criteria so that participants reflect those seen in community settings; and (d) incorporate real‐world comparison conditions, such as “services as usual” rather than no treatment or control groups. In addition to these recommendations, community psychologists have stressed the importance of maximizing the safety and confidentiality of clients, taking into account agencies’ competing priorities, and considering agencies’ and communities fluctuations in resources and funding (Nnawulezi, Sullivan, Marcus, Young, & Hacskaylo, 2019; Sullivan, 2011). While all of these recommendations are laudable and pragmatic evaluations are certainly not novel to community psychologists, the field lacks concrete examples of how such principles can be put into practice. We especially lack information on the decision‐making processes that go into designing a pragmatic evaluation. This manuscript attempts to fill the research‐to‐practice gap by providing one example of a pragmatic evaluation designed to maximize both methodological rigor and ecological validity. The example involves evaluating The Domestic Violence Housing First (DVHF) Model—an innovative, community‐based model intended to help survivors of intimate partner violence obtain safe and stable housing. This large‐scale demonstration project is currently ongoing in Washington state, in collaboration with the Washington State Coalition Against Domestic Violence. The goal of the longitudinal evaluation is to examine the extent to which the DVHF model leads to long‐term safety, housing stability, and well‐being for domestic violence (DV) survivors and their children, compared to survivors receiving services as usual. Before describing the development of the study design in more detail, we first provide a brief description of the DVHF model.

## The Domestic Violence Housing First Model

The DVHF model was designed to enhance the safety and housing stability of DV survivors. DV is a leading cause of homelessness for women and children, and, in turn, lack of stable housing increases women’s risk of victimization (Pavao, Alvarez, Baumrind, Induni, & Kimerling, 2007). Unfortunately, little evidence exists to date about effective strategies to assist women as they work to avoid homelessness while freeing themselves and their children from the abuse of partners and ex‐partners (Baker, Cook, & Norris, 2003; Bassuk, Dawson, & Huntington, 2006). Even less is known about effective strategies for men, gender diverse or trans survivors.

The DVHF model is an adaptation of the Housing First model, which has empirically demonstrated the importance of helping homeless individuals obtain stable housing as quickly as possible—regardless of whether they have first addressed other issues such as substance abuse or mental health treatment (Greenwood et al., 2020; Padgett, Henwood, & Tsemberis, 2016; Tsemberis, 2010). However, the Housing First model was originally created to address the needs of chronically homeless, single adult men, necessitating its adaptation for work with DV survivors. While additional information about this adaptation can be found in Sullivan and Olsen (2016), the DVHF model involves three pillars—(1) survivor‐driven, trauma‐informed, mobile advocacy; (2) flexible financial assistance; and (3) community engagement—to help DV survivors obtain safe and stable housing.

### Survivor‐Driven, Trauma‐Informed, Mobile Advocacy

A critical component of the model is that advocates focus on addressing the needs identified by survivors rather than on pre‐determined needs promoted by agencies. Advocates are also mobile, meeting survivors where it is safe and convenient for them, and advocacy continues as long as survivors need support. Advocates are aware of the myriad ways that abusers sabotage survivors’ economic and housing stability—even after the relationship has ended—and they mobilize multiple resources and community supports to prevent or counter these abusive activities. Based on prior evidence that mobile advocacy results in less reabuse and increased well‐being of DV survivors and their children (Bybee & Sullivan, 2002; Sullivan & Bybee, 1999; Sullivan, Bybee, & Allen, 2002), advocates are proactive and creative, accompanying survivors to housing appointments, acting as liaisons with landlords, and negotiating leases.

Further, given the traumatic nature of DV, as well as the likelihood that DV survivors have also experienced other lifetime traumas such as child abuse and sexual abuse (Kennedy et al., 2012), a critical tenet of DV Housing First is to engage in trauma‐informed practice. These practices include the following: (a) establishing emotional safety; (b) restoring choice and control; (c) facilitating survivors’ connections to community supports; (d) supporting positive coping strategies; (e) responding to identity and context; and 6) building strengths (Goodman, Fauci, Sullivan, DiGiovanni, & Wilson, 2016; Harris & Fallot, 2001).

### Flexible Financial Assistance

Many survivors need not only proactive advocacy to obtain safe and stable housing, but also temporary financial assistance to get back on their feet. They may need financial assistance with issues viewed as directly related to housing: a security deposit and temporary rental assistance, help clearing up rent arrears (often intentionally created by the abuser), or help with utility bills. Often, though, survivors need funds that may not be viewed by others as impacting housing but that advocates recognize as being critical to housing stability: for example, help repairing their cars so they do not lose their jobs, help expunging a prior conviction that is preventing them from obtaining government‐funded housing, or help repairing bad credit (often destroyed by the abuser). Funds are targeted to support survivors so they can rebuild their lives, including covering childcare costs, transportation, school supplies, work uniforms, and permits required for employment, as well as other individualized concerns (Mbilinyi, 2015; Sullivan, Bomsta, & Hacskaylo, 2019).

### Community Engagement

Advocates also proactively engage those people in the community who can help support the safety, stability, and well‐being of survivors. This includes engaging with healthcare professionals, law enforcement and the legal systems, educators and school administrators, religious and spiritual leaders, and others (Sullivan & Olsen, 2016).

## Large‐Scale DVHF Evaluation

Although there is limited evidence regarding its impact, the DVHF model continues to proliferate nationally (for more information with regards to this model see: https://wscadv.org/projects/domestic‐violence‐housing‐first/). This widespread interest is due in part because this model incorporates evidence‐informed and evidence‐based components, and also because it resonates with what practitioners believe to be effective practices. Rigorous evidence regarding this model is urgently needed as implementation efforts continue to rise. To address this call, our research team was invited to design a longitudinal evaluation that could examine whether and how the DVHF model leads to housing stability, safety, and well‐being for survivors and their children over time.

## Choosing the Most Appropriate Design for this Evaluation

A common challenge in real‐world evaluations is choosing a design that is rigorous yet practical. In determining how best to test the outcomes of the DVHF approach in real‐world settings, a number of study designs were considered. Our research team (including the Housing Director of the Washington State Coalition Against Domestic Violence) visited programs, examined service delivery records, and talked with program staff to fully understand how services were offered within each agency and which study design would be the most feasible.

We initially examined whether an RCT would be practicable, considering that it was clear that not all survivors eligible for DVHF were receiving the model. This option was rejected for a number of reasons. First, we agreed with agencies that randomizing survivors into particular services posed ethical problems—we could not jeopardize survivors’ safety for the purpose of research. Further, resource availability within each agency was quite unpredictable—for example, agencies do not tend to know when a shelter bed will open up, when a permanent housing voucher will become available, or when affordable housing has an opening. Finally, there is ongoing staff turnover, which impacts the amount of time that can be provided to survivors.

Even if the other factors precluding the success of using an RCT approach were not evident, the likelihood of random assignment failing was high, which would have jeopardized the entire study (Brown et al., 2020). Although RCT designs can work well if the investigators have control over both the intervention being delivered and the randomization process, expecting community members to randomize clients into conditions is fraught with problems (Gondolf, 2010). An early example of randomization difficulties was found in an NIJ‐funded RCT study of police officer response to DV (Berk, Smyth, & Sherman, 1988), and more recent examples of challenges randomizing community‐based samples have been noted in the United States Department of Housing and Urban Development (HUD)‐funded Family Options Study (Gubits et al., 2016) and a recent HUD‐funded Housing First evaluation (Brown et al., 2020).

Another important consideration in choosing the research design centered around how service delivery was determined. The evaluation team carefully examined whether survivors were receiving services based on their needs or whether services were provided based on agency capacity. Had agencies targeted different services to different situations, this would have represented a serious validity threat to following all survivors for a specific period of time who receive agency services. After examining records and talking specifically with direct service staff about a number of recent unstably housed or homeless clients (to ascertain what the client wanted from the agency and what they were offered), it became clear that none of the agencies were consistently matching survivors to specific services based on need. While they all purported to be survivor‐focused in theory, they also acknowledged that due to limited resources (e.g., shelter beds, transitional housing units, flexible funding), it was often the case that survivors received “what was available at the time.”

In the end, the research team decided to capitalize on the reality that no DV victim service program can adequately meet the needs of all survivors who seek assistance from them. At each agency, it was clear that not all survivors who were eligible for DVHF were actually receiving it. As detailed earlier, there are many times that shelters are full, advocates are overcommitted or unavailable, and/or flexible funding is limited or nonexistent. These fluctuations are not predictable and do not lend themselves to randomization. Sometimes survivors are able to receive all of the services they need, but other times they either receive too little or they receive assistance that does not match their needs. Based on these initial, extensive conversations with program staff, we anticipated that at least 50% of survivors in the study would receive some level of mobile advocacy and/or flexible funding. The team decided that systematically inviting all eligible survivors into the study across a period of time would ensure capturing natural variability in service delivery, and therefore result in the most generalizable and meaningful findings.

The design chosen for the current study maintains adequate internal validity while maximizing external validity, and will do what many studies in the past have failed to do: it will carefully document the details about what survivors receive over time, not just from the agency they were recruited from, but from other community sources as well. We are documenting the exact amount of money (if any) survivors receive through flexible funds, as well as the amount of time they spend with their advocate(s). Additionally, we are examining when such activities happen and how they affect survivors’ safety, housing stability, and well‐being over time.

## Multi‐Method, Multi‐Source, Quasi‐Experimental Longitudinal Design

Real‐world evaluations are often limited by gathering information from only one source of data or through one method, which can impact the breadth and depth of information considered in assessing the value of a program. In order to gain the most comprehensive information about the DVHF intervention over time, the research team decided to employ multi‐source, multi‐method measurements. This involves collecting data from (1) agency clients, (2) their advocates, and (3) agency records. Details regarding what we decided to collect, and from which data source, are presented next.

Data are being collected from clients, staff, and written records from the five DV agencies participating in the longitudinal study. They were chosen because they are part of the DVHF demonstration project funded through the Washington State Coalition Against Domestic Violence by the Bill & Melinda Gates Foundation, they work with a large enough number of clients annually to provide the desired sample size, they are similar in structure to each other and to many programs across the country, and they have the infrastructure capacity to participate in a rigorous evaluation study. Two agencies are located in urban areas and three are located in more rural areas so that we can examine how this intervention may differ depending on geographical context. At the time of this writing, all baseline data have been collected and follow‐up data collection is in progress.

### Interviews with Survivors

Survivors are being interviewed every six months over a period of two years, beginning when they first contact a participating agency for help. Over the course of study recruitment (which has now ended), all clients who sought services from the agencies were told about the evaluation if they were recent DV survivors, and were either homeless or at immediate risk of becoming homeless. Advocates within each agency were carefully trained to identify eligible new clients and to refer them to hear more about the study from an IRB‐trained research team member. Survivors’ emotional safety needs were prioritized over their hearing about the research study, so some survivors were not invited into the study until after a couple weeks had passed. However, the goal was to invite all clients within one week of their contacting an agency for help. Site coordinators from the evaluation team stayed in frequent contact with agencies to ensure that all eligible clients heard about the project.

After hearing more about the study from an evaluation team member and agreeing to participate, 406 survivors were interviewed in person in a private location of their choosing (usually their home, the agency, or a public library or community center). Baseline interviews assessed historical context as well as current needs of survivors and what they hoped to receive from the agency. Follow‐up interviews, which are still ongoing, include specific questions about DVHF components and dosage. These are asked of all study participants in order to ascertain who has not received sufficient “dosage” of DVHF. The fidelity measure examines (a) needs related to safety, housing stability, and economic stability, (b) specific activities they engaged in with their advocates to meet those needs, (c) amount of time spent with advocates, (d) the extent to which the survivor guided intervention activities, (e) effectiveness of efforts, and (6) their satisfaction with various aspects of the program.

Additional measures included in participant interviews over time pertain to how trauma‐informed and culturally relevant any services received from the participating programs are perceived. Interviews also contain questions about a variety of contextual factors that could impact safety and housing stability over time (e.g., substance abuse, social support, employment) and that will help capture the range of supports and services received by those not receiving DVHF. Finally, questions about children’s academic and socio‐emotional well‐being are included for those people parenting minor children.

### Designing Trauma‐Informed, Culturally Responsive Interviews

When creating the interviews, the research team needed to balance the desire to gather comprehensive information with the need to keep interviews reasonable in length. We had to carefully weigh the value of each measure to the overall study goals, and also consider language needs, literacy, interviewee fatigue, and risks of retraumatization. Respectfully conducting interviews with multiply marginalized individuals requires not just strong interviewing competencies, but also skills in effective listening, crisis intervention and safety planning, and cultural humility (Campbell, Adams, Wasco, Ahrens, & Sefl, 2010; Sullivan & Cain, 2004).

Unlike the idea of “cultural competence,” which suggests one can achieve a final state of cultural proficiency, cultural humility involves a commitment to ongoing self‐reflection and lifelong learning (Fisher‐Borne, Cain, & Martin, 2015; Garneau & Pepin, 2015). Designing and implementing research from a position of cultural humility necessitates questioning one’s own assumptions and biases as they have been shaped by specific sociopolitical locations, while maintaining genuine curiosity about and studying others’ cultural backgrounds and identities (Yeager, & Bauer‐Wu, 2013). When designing the current study, this stance involved forming a diverse research team, learning as much as possible about the experiences of DV and housing instability among different cultural groups (e.g., migrant farmworkers, indigenous peoples) when designing interviews, and committing to the values of linguistic justice, which includes ensuring that participants have the opportunity to communicate in their preferred language and that their responses will be accurately interpreted (Avineri, Graham, Johnson, & Riner, 2018).

We have found that the commitment to conducting trauma‐informed, culturally responsive interviews also requires extensive supervision of interviewers over time, which includes dedicated time for them to focus on their implicit biases and emotional needs. Our team engages in weekly “processing” meetings where interviewers are encouraged to reflect on their biases, concerns, questions, and successes within the project. These strategies have resulted in high retention of interviewers over the course of the study, and we believe it has contributed to the retention of research participants as well. A number of participants have commented that interviewers are incredibly compassionate, and have noted that this keeps them connected to the study.

In keeping with the values of community‐based research, we committed to conducting all of our interviews in person, in the community, in spaces that were easily accessible, comfortable, and safe for survivors. Depending on participant preference, this means that interviews occur in private rooms in local libraries or community centers, at the participating DV agency, or in the participants’ homes. Interviews are never conducted within an academic space where survivors might feel othered or unwelcome. The original plan was that interviews would only be conducted by phone if the participant moved out of the area or if they preferred this mode. However, after the COVID‐19 outbreak, all interviews had to be conducted by phone or Zoom, and this will continue until the pandemic ends.

Finally, we made sure to proactively offer and provide money to cover transportation or childcare when such issues interfered with the participant’s ability to attend an interview. We believe these decisions have improved level of engagement as well as data quality, as participants have commented on these conveniences as being important to their willingness to stay engaged in the study.

### Maximizing Retention

In a longitudinal evaluation, retention is of utmost importance since the people lost to retention efforts over time tend to differ in important ways from those who are retained. To maximize response rates at each time point, we are using procedures similar to those that resulted in a 94+% retention rate over two‐year follow‐up in the first author’s prior study involving DV survivors (Sullivan, Rumptz, Campbell, Eby, & Davidson, 1996). The first phase of the retention process consists of “setting the stage” by promoting trust with participants, as well as implementing reminders for future interviews, providing a phone line for participants to call or text if necessary, and clarifying compensation (in this case, $50 per interview). The second phase consists of implementing proactive and creative retention strategies (e.g., visiting participants at home when requested). The final phase involves using social network and community‐oriented strategies to contact participants. Participants are contacted every three months in order to ascertain if their contact information has changed or is expected to change, and we ask for contact information for anyone in their lives who is likely to know how to find them over time, as well as permission to contact these individuals if necessary. All retention strategies were designed to ensure participants’ safety and confidentiality.

### Advocate Surveys

Pragmatic evaluations benefit if they include multiple sources of data to assess a program. For this study, we believed it was critical to include the voices of the advocates providing DVHF services. During the 6‐month interview, study participants are asked to provide the name of the primary advocate they worked with, in order for us to obtain additional information from them. Collecting data from advocates will provide us with critical information about which the survivors may be unaware. Agency clients rarely know everything that happens “behind the scenes” in an agency, as advocates may spend hours seeking assistance from colleagues, negotiating with those in control of resources (e.g., landlords), and researching how they might be most helpful to clients. Agency clients have limited information, and agency staff have limited information—collecting data from both parties will provide a more comprehensive picture of the DVHF model.

Advocates are not told what their clients reported during any interview, but when they are nominated by a participant, they are invited to complete a brief online survey about their work on behalf of that particular client. In addition to providing basic demographic and work background about themselves, advocates report on the various housing barriers that their client has faced, and what services they provided to stabilize the client’s housing status, safety, and well‐being. They are also asked to predict the likelihood of the survivors’ housing stability in the next six months, as well as specific services and activities the survivor may require in the near future to secure and sustain safe and affordable housing. Information from advocates is collected using a web‐based computer‐assisted self‐interview (CASI) platform. This method was chosen so that advocates could complete the brief surveys at a time convenient to them, in a manner that is private and confidential.

### Agency Records

Agencies often collect detailed data about the services they provide, and gathering such information can strengthen a comprehensive evaluation. We also believed it was important to gather any data from agencies that we could, in order to minimize having to ask the clients themselves for such information. The addition of agency records to our data plan has two overarching benefits: (1) it drastically reduces the burden on participants to provide information that is available elsewhere, and (2) it provides us with valuable information about participants who may not complete all interviews over the five data collection time points.

All of the participating agencies are carefully documenting a number of factors that we have permission to obtain. They are providing service start and end dates for clients participating in the study, and documenting which services are provided to them over time. All of the participating agencies also systematically track their use of flexible funding. Each program tracks when a survivor receives funds, how much they receive, and what specifically funds are spent on. These records allow us to link documentation of all services provided to the survivor at each agency throughout their two years as study participants. In addition, we will have access to flexible funding records for each individual participant over the study period, even if they are lost to the study over time.

In addition to providing information about individual clients, agencies are also documenting critical contextual information about their agency resources each month. They are reporting, by month, how many advocates they have available to provide DVHF, the average caseload of DVHF advocates, number of days they have shelter beds or transitional housing space available, how much money the agency has on hand to provide flexible funding, and the number of permanent housing vouchers they had available in the prior month. These data will help us consider a number of important contextual factors that can impact the delivery of the model (e.g., staff caseload, availability of funds), as well as client outcomes.

## Choosing the Appropriate Analytic Plan

Given the multiple sources and multiple methods of data being collected over time in this study, it was critical that we select an analytic plan well‐suited to handling such data. A number of analytic options are especially suitable for complex, longitudinal data; however, some important decisions needed to be made prior to analysis. Our overarching hypothesis is that survivors who receive the DVHF model will have greater safety, housing stability, economic stability, and well‐being over time compared to survivors receiving “services as usual.” Therefore, one of the most important decisions centered on how to measure “receiving DVHF services” vs “standard DV services.” Categorizing the types of services received at each time point is being drawn from the extensive data collected from survivors, advocates, and agency records. As data continue to be collected, if the distribution of services cannot be clearly dichotomized into these two categories, we decided to use latent class analysis (LCA) to determine groups. LCA is a person‐centered approach that assumes sample heterogeneity and allows for the examination of conceptually important and distinguishable subgroups (i.e., classes), whereas variable‐oriented analytic approaches assume homogeneity within populations (Howard & Hoffman, 2018).

Given that we expect DV survivors from different life circumstances to have different trajectories, the person‐centered LCA approach will also be used to examine change over time among subgroups of participants. In addition, latent transition analysis (Collins & Lanza, 2010) will allow us to examine which variables impact change in class over time—for example, whether and how social support or employment might predict participants transitioning from high housing instability to low housing instability.

Another important pre‐analysis decision was the choice to use propensity scores to adjust for pre‐existing differences that may exist between individuals who received DVHF and those who received standard services (Lanza, Moore, & Butera, 2013). Although we believed that much of the variation in services provided would likely be related to fluctuations in agency resources (e.g., advocates’ caseloads, availability of flexible funds), some variation in services received may be related to characteristics of survivors themselves (e.g., their history of homelessness, barriers to stable housing, family characteristics). Because baseline differences may affect outcome trajectories (i.e., it may not be reasonable to assume that outcome trajectories will be parallel despite survivors starting at different baseline levels), it may be necessary to adjust for baseline differences in order to reduce this potential source of bias (Abadie, 2005). The propensity model will be carefully examined to ensure that it is effective in balancing the groups on all baseline covariates (Austin, 2011; Austin & Stuart, 2015).

Finally, to test the effect of DVHF on each of the major outcomes, we chose to use mixed effects longitudinal regression, also known as longitudinal multilevel modeling or longitudinal MLM (Hedeker & Gibbons, 2006; Singer & Willett, 2003). This method has numerous advantages and will allow us to simultaneously characterize the overall trajectory of change on each outcome from pre‐intervention through 24‐month follow‐up and test for differences in the trajectories of change between those who received DVHF and those who received “standard services.” MLM will also allow us to test for outcome differences between service types at each observation point.

## Study Limitations

We are excited about the breadth and quality of data we expect to obtain through this project, but the study design is not without its limitations. For example, the bulk of our data come from lengthy interviews with survivors, and there is always a degree of recall bias when interviewing participants about their experiences over the prior six months. Further, within that six‐month time frame we are not capturing temporality. While we may know that within that time frame a survivor experienced violence and job loss, for example, we will not know which preceded which. Although the Life History Calendar method or a similar procedure could have captured this (Morselli et al., 2016), the interview was already over an hour long as created, so we chose to forego this much more time‐intensive option.

This design is also sparse on qualitative data collection. We know that contextual factors will impact participants’ stability and well‐being over time, regardless of whether or not they received DVHF, and having more extensive qualitative data would provide even more depth to our understanding. We do intend to conduct targeted qualitative studies within the larger study to help us interpret and further explore quantitative findings. For example, a number of participants have reported that they are financially supporting family members in another country. We may do qualitative interviews with these individuals to better understand how this impacts their financial well‐being, housing, safety, and health. However, across the entire sample of 406 participants, we have limited qualitative data.

Although we have a diverse sample, we were not able to capture the perspectives of some populations that continue to be underrepresented in research. For example, we were not able to reach as many indigenous survivors as we had hoped, and the sample is almost entirely comprised of cisgender women. We were also only able to offer interviews in Spanish or English, limiting participation of survivors who preferred to be interviewed in other languages. On the other hand, the heterogeneity of our sample can also be viewed as a limitation. If participants have extremely varied life histories, circumstances, and experiences over the two years, it will be more difficult to clearly identify generalizable impact of DVHF on survivors’ safety and housing stability. Finally, we recognize that this study design was possible because we were fortunate to have received significant public and private funding. Not all of the methodological components described here would be possible with a more restricted budget.

## Conclusion

Community psychology has an explicit commitment to conducting real‐world research that takes context and structures into account. Here we provided one tangible example of the decision‐making processes that go into designing such projects. We offered this example as an illustration of how a pragmatic evaluation, utilizing multi‐source data within a multi‐method design, and that capitalizes on analytic techniques especially appropriate for real‐world, longitudinal data, has maximized the likelihood of producing findings that community members will find to be persuasive and useful to their work. While this study focused on an intervention for unstably housed DV survivors, we believe many components are generalizable to other studies of nonprofit agencies working with multiply marginalized clients.

Real‐world evaluations often need to look beyond the RCT as being the most rigorous and accurate study design (Goodman et al., 2018). Quasi‐experimental studies may not only be more palatable to community partners in many situations, they can actually be far more rigorous designs than RCTs with regard to examining how real‐world interventions are impacting multifaceted individuals living in complex environments (Schwandt, 2015). They allow for incorporating the “messiness” of people’s experiences and contexts into studies, which will result in data that actually reflect how interventions work with diverse individuals across a variety of communities. We do not present this model as a benchmark or gold standard, but rather as one concrete example offered in the spirit of sharing such processes so we can learn from each other’s advances, as well as limitations.
